# Stress Reduction Through Listening to Indian Classical Music During Gastroscopy

**DOI:** 10.1155/DTE.4.191

**Published:** 1998

**Authors:** M. Raj Kotwal, C. Z. Rinchhen, Vishwajeet V. Ringe

**Affiliations:** 1 Sir Thutob Namgyal Memorial Referral Hospital Gangtok Sikkim India; 2 National Information Centre Tashiling Gangtok Sikkim 737101 India; 3 Shunyata Tibet Road Gangtok Sikkim 737 101 India

## Abstract

The purpose of this study was to examine the effects of music on elevated state of anxiety as many patients become stressed and anxious during diagnostic procedures. The study was conducted on 104 consecutive patients undergoing GI endoscopy for various reasons. Patients were randomly assigned to two groups regardless of sex, age and underlying disease. One group of 54 patients were made to listen to a recorded Indian classical instrumental music before and during the procedure, while the other group of 50 patients did not. Blood pressure, heart rate and respiratory rate were recorded at the beginning of consultation and end of procedure. Perception of procedure using a three point attitude scale was assessed. Our results indicate that the background Indian classical music is efficacious in reducing psychological distress during a gastroscopic examination. We suggest that music could be applied to other medical situations as well, which tend to generate undue psychological stress and anxiety. Music, as a familiar personal and culture medium, can be used to ease anxiety, to act as distractor, to increase discomfort and pain threshold.


*Diagnostic and Therapeutic Endoscopy*, Vol. 4, pp. 191–197
Reprints available directly from the publisher
Photocopying permitted by license only

© 1998 OPA (Overseas Publishers Association)
Amsterdam B.V. Published under license
under the Harwood Academic Publishers imprint,
part of The Gordon and Breach Publishing Group.
Printed in Singapore.

### Stress Reduction through Listening to Indian Classical Music during Gastroscopy

M. RAJ KOTWAL[a,*], C.Z. RINCHHEN[a] and VISHWAJEET V. RINGE[b]

[a] *Sir Thutob Namgyal Memorial Referral Hospital, Gangtok, Sikkim, India;*
[b] *National Information Centre, Tashiling, Gangtok, Sikkim 737101*

*(Received 21 January 1997; In final form 29 September 1997)*

**The purpose of this study was to examine the effects of music on elevated state of anxiety as many patients become stressed and anxious during diagnostic procedures. The study was conducted on 104 consecutive patients undergoing GI endoscopy for various reasons. Patients were randomly assigned to two groups regardless of sex, age and underlying disease. One group of 54 patients were made to listen to a recorded Indian classical instrumental music before and during the procedure, while the other group of 50 patients did not. Blood pressure, heart rate and respiratory rate were recorded at the beginning of consultation and end of procedure. Perception of procedure using a three point attitude scale was assessed. Our results indicate that the background Indian classical music is efficacious in reducing psychological distress during a gastroscopic examination. We suggest that music could be applied to other medical situations as well, which tend to generate undue psychological stress and anxiety. Music, as a familiar personal and culture medium, can be used to ease anxiety, to act as distractor, to increase discomfort and pain threshold.**

*Keywords:* Stress reduction, Music, Gastroscopy

### INTRODUCTION

A gastrointestinal endoscopy service requires a suitable ambient environment. Many patients fear GI endoscopy. Natural anxiety may be aggravated by horror stories from friends or inappropriate remarks by endoscopy staff. Good technique is essential and some medication is usually given, but the acceptability of endoscopy is also crucially dependent upon careful, sympathetic explanation and a reassuring friendly atmosphere at the time of reception as well as during the examination. Endoscopy can become such a routine to the doctors and nurses concerned that patients' natural anxieties may be ignored and thereby increased [1]. An extensive review of literature on stress and health has been presented by Kasl [2]. Dobson [3] has found it difficult to give an adequate definition of stress which would be acceptable to all. However, Lazarus [4] through his writing in International

* Corresponding author. Shunyata, Tibet Road, Gangtok-737 101, Sikkim, India. Tel.: 91-3592-22997; 91-3592-25100; 91-3592-23121. Fax: 91-3592-22707.

Encyclopedia of the Social Sciences in 1968 remarked that stress suggests excessive demands made on man and animal that produce disturbances of physiological, social and psychological systems.

The control of stress by drugs like tranquilizers has the disadvantage that it is not a satisfactory long term answer to the problem of severe stress [5]. Music stands for the mystical expression of life cycle's celebrations, birth, death, renewal of seasons, hunting and rituals of passage. It serves on other familiar conjunctures, such as the dentist chair [6], waiting rooms, on the telephone, and air travel [7], helping us to relax or increase our patience. However, music is not for everyone at all times. With each individual, its significance varies according to the moment and the situation [8]. Our GI set up has been using Indian classical music as an adjunct in GI endoscopy since 1983. We decided to evaluate the scientific and therapeutic possibilities in this experiment.

### MATERIALS AND METHODS

The study was conducted on 104 consecutive patients undergoing GI endoscopy for various reasons. Patients were randomly assigned to two groups regardless of sex, age or underlying disease. One group of 54 patients listened to the recorded classical Indian instrumental music and the other of 50 patients did not. Blood pressure, heart rate and respiration were recorded at the beginning of consultation and termination of endoscopy. Blood pressure and heart rate were recorded in all the patients in music as well as without music group. Respiratory rate was recorded in 34 and 29 patients in music and without music group respectively. The group assigned to music was made to listen to music for 10 min before the procedure and throughout the procedure, while the other group without music waited. No sedation or topical anesthesia was used in any group. We also evaluated the perception of procedure using a three point attitude scale comprised of the following rankings: (A) a mild uncomfortable procedure which I could repeat if so advised; (B) a moderately uncomfortable feeling, but still can undergo if advised; (C) a severely uncomfortable experience and would not like to repeat again [9].

### RESULTS

Using paired $T$-test in the group of patients who were made to listen to music, there is statistically significant difference in blood pressure, systolic, diastolic, heart rate and respiratory rate – Table I. The group of patients who were not made to listen to the music had statistically significant difference

TABLE I Patients exposed to the music (difference of values)

| | BP-S | BP-D | H-RATE | R-RATE |
|---|---|---|---|---|
| SUM $S$ | 664.00 | 412.00 | 163.00 | 162.00 |
| NO. $N$ | 54.00 | 54.00 | 54.00 | 34.00 |
| S.S. | 17200.00 | 7000.00 | 3633.00 | 2474.00 |
| MEAN $M$ | 12.30 | 7.63 | 3.02 | 4.76 |
| SD | 13.06 | 8.53 | 7.70 | 7.18 |
| $T$-VALUE | 6.92 | 6.57 | 2.88 | 3.87 |
| $T$ .025, $N$-1 | 2.02 | 2.02 | 2.02 | 2.03 |
| H0 (NULL) | R | R | R | R |

BP-S: Systolic Blood Pressure; BP-D: Diastolic Blood Pressure; H-RATE: Heart Rate Per Minute; R-RATE: Respiratory Rate Per Minute.

*Conclusion*: From the above result, it is clear that there is a significant difference in all the four parameters (BP-S, BP-D, H-RATE, R-RATE) in the difference of values at the beginning and end of examination, when patients were exposed to the music.

*Note*: R: Rejected.

only in systolic blood pressure. In fact respiratory rate increased – Table II. When analysis of data for patients between two groups was compared there is again statistically significant difference in the three parameters, blood pressure, systolic, diastolic and respiratory rate – Table III. All the four parameters recorded at the beginning of the consultation and at the end of procedure are shown in Figs. 1 and 2. It is therefore concluded that there is statistically significant difference in (with and without music) two groups of patients. Perception of procedure using a three point attitude scale has been shown in Fig. 3. The number of patients who reported decrease in distress was markedly higher in music group than the control group.

### DISCUSSION

Subjects undergoing upper gastrointestinal endoscopy develop high anxiety levels [10]. In routine cases, local anesthesia of the pharynx is performed by lidocaine spray [11], and a subcutaneous injection of an anticholinergic agent (0.5 mg atropine sulphate [12], 20 mg valetamate bromide or 40 mg scopolamine butyl bromide) is sufficient. In particularly nervous cases an additional intravenous injections of 7.5–15 mg pentazocine is administered [13]. In cases exhibiting fear, or in subjects unable to comprehend the procedure 5–10 mg of diazepam or 35–50 mg meperidine hydrochloride is injected intravenously for sedation immediately

TABLE II Patients not exposed to the music (difference of values)

| | BP-S | BP-D | H-RATE | R-RATE |
|---|---|---|---|---|
| SUM, *S* | 218.00 | 76.00 | 134.00 | (92.00) |
| NO., *N* | 50.00 | 50.00 | 50.00 | 29.00 |
| S.S., SS | 7468.00 | 4046.00 | 6244.00 | 1464.00 |
| MEAN, *M* | 4.36 | 1.52 | 2.68 | (3.17) |
| SD, SD | 11.53 | 8.96 | 10.96 | 6.47 |
| *T*-VALUE | 2.67 | 1.20 | 1.73 | (2.64) |
| *T*.025, *N*−1 | 2.01 | 2.01 | 2.01 | 2.05 |
| H0 (NULL) | R | A | A | R |

BP-S: Systolic Blood Pressure; BP-D: Diastolic Blood Pressure; H-RATE: Heart Rate Per Minute; R-RATE: Respiratory Rate Per Minute.
*Conclusion*: From the above result, it is clear that there is a significant difference in the two parameters (BP-S, R-RATE) in the difference of values at the beginning and end of examination, when patients were not exposed to the music, while R-RATE has significantly increased at the end of the examination.
*Note*: R: Rejected; A: Accepted.

TABLE III Analysis of data for patients between two groups (with and without music)

| | BP-S | BP-D | H-RATE | R-RATE |
|---|---|---|---|---|
| SD-com | 12.35 | 8.74 | 9.41 | 6.86 |
| *T*-value | 3.27 | 3.56 | 0.18 | 4.57 |
| *T*.025, *n*1 + *n*2−2 | 1.60 | 1.60 | 1.60 | 2.00 |
| H0 (NULL) | R | R | A | R |

BP-S: Systolic Blood Pressure; BP-D: Diastolic Blood Pressure; H-RATE: Heart Rate Per Minute; R-RATE: Respiratory Rate Per Minute.
*Conclusion*: From the above result, it is clear that there is a significant difference in the three parameters (BP-S, BP-D and R-RATE) in the two groups of patients. It is therefore concluded that there is a statistically significant effect of music on systolic, diastolic blood pressure and respiratory rate in the two groups of patients.
*Note*: R: Rejected, A: Accepted.

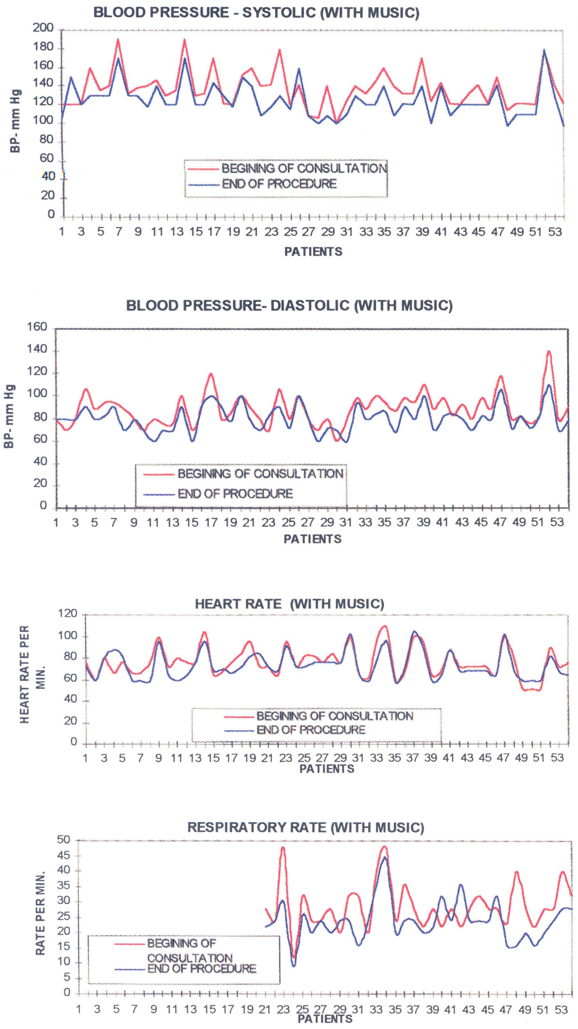

FIGURE 1

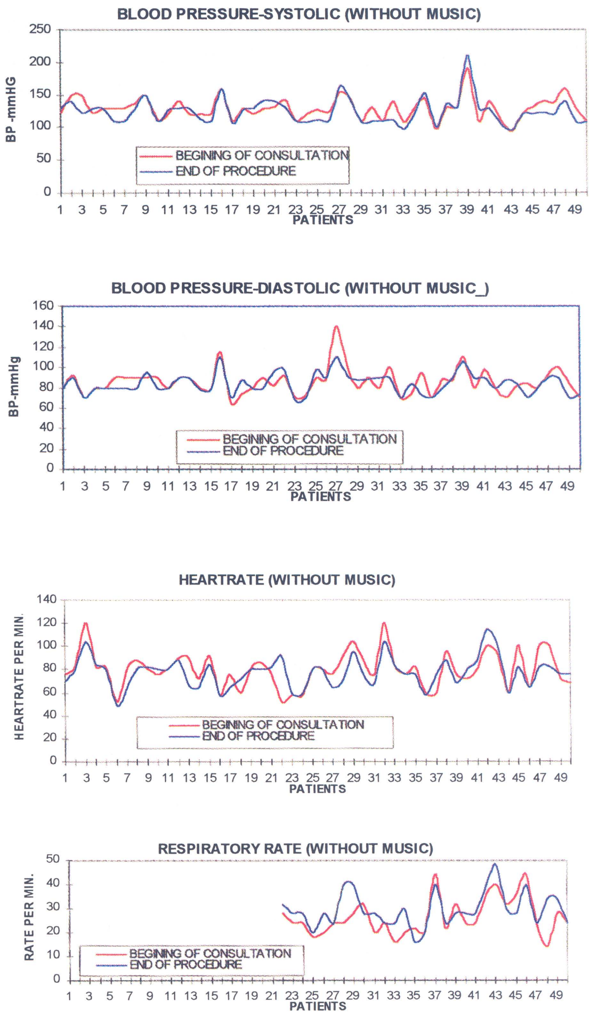

FIGURE 2

prior to the procedure [14,15]. Opinions and practices concerning analgesia and sedation vary widely between different centers and cultures. Most units use medication, but some experts rely solely on good technique, rapport and speed. Endoscopy without medication is better tolerated by older patients than by younger ones. It is safer in patients with pulmonary problems. Endoscopy is also easier to organize when sedation is avoided; there is no need for formal recovery, and fit patients can drive or return immediately to work or play [1]. Prosperous societies have chronic illnesses related to stress and life-style. "Anywhere from 60% to 90% of visits to

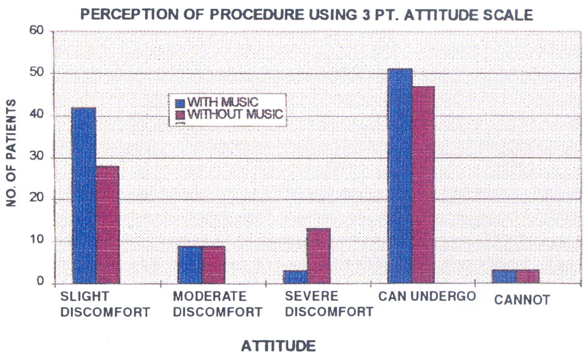

FIGURE 3

doctors are in the mind–body, stress related realm" asserts Dr. Herbert Benson, President of the mind/body medical institute of Boston's Deaconess Hospital and Harvard Medical School. It is a triumph of Medicine that so many of us live long enough to develop these chronic woes, but, notes Benson, "traditional modes of therapy – Pharmaceutical and surgical – don't work well against them". Benson won international fame in 1975 with his best-selling book *The Relaxation Response*. In it he showed that patients can successfully battle a number of stress-related ills by practicing a simple form of meditation. The act of focusing the mind on a single sound or image brings about a set of physiological changes that are the opposite of the "fight- or flight response." With meditation heart rate, respiration and brain waves slow down, muscles relax and the effects of epinephrine and other related hormones diminish [16]. Basically, originally, music was used for meditation, in particular Indian music developed as a method for meditation. Gandharva Veda (a branch of the vast vedic literature loosely translated as "the knowledge of musical tones"). Veda literally means "science" or "knowledge", referring to complete knowledge of manifest and unmanifested creation. Gandharva music originated many centuries ago in ancient India; today its rules still form the basis for the long, beautiful improvised ragas that Indian musicians play. Gandharva Veda embodies some very sophisticated techniques for changing physiology. Music is more than "soothing" or "rousing". Why do we listen to music in the first place? For pleasure, of course, but all pleasures change the body some way.

Ordinarily we do not measure our blood pressure to see how Bach or Mozart might be affecting it, but if you want to lower blood pressure, listening to soft, slow classical music is considered very good medicine. Gandharva texts have specified which ragas are appropriate for morning, noon, evening and other times of the day. When properly played, Gandharva melodies are said to have universal effects. Our bodies are responding with changes that mirror the varying rhythms of nature, it is not just your pulse that comes down in the evening, after all; every plant and animal reacts according to its own evening cycles, too. Gandharva music embodies the fundamental vibrations that pulsate through nature at every moment [18]. Music has been used for pain [19–21] relaxation [22–25], gastroscopy [26], internal medicine [27,28], epidural anesthesia [29,30], psychiatric conditions [31–37], diagnostic procedures [38,39], brain damage [40–43], surgical holding area [45–47], in the care of new born [48,49], sleep disturbances in the elderly [50], palliative care [51,52], to reduce anxiety in myocardial infarction [53–56], disabilities [57], cancer related pain [58–60], harmony for change [61–63], Alzheimer's [64], in intensive care management [65–67] and stress reduction [68,69]. The number of patients who reported distress tended to decrease in the music group. Our results indicate that the background Indian classical instrumental music is efficacious in reducing psychological distress during a gastroscopic examination, mainly by curtailing the patients' subjective sense of examination time and relaxation. We suggest that sedation could be administered at the optimum requirement, either as per demand in a particular clinical situation, or not at all. Music could be applied to other medical situations, which tend to generate undue psychological stress and anxiety. Music, as a familiar personal and culture medium can be used to ease anxiety, to act as a distracter, to increase discomfort- and pain threshold.

### *Acknowledgements*

The authors express gratitude to patients and staff of the Department of Gastroenterology. Sincere gratitude is expressed to National Informatic Centre, Sikkim, Indian Statistical Research Institute, Delhi, Mr. Karma P. Bhutia, Superintending Engineer, and Mr. P.R. Nambiar for their secretarial assistance on computers.

### *References*

[1] Contton, P.B. and William, C. *Gastrointestinal Endoscopy*, Third edition, Blackwell Scientific, 1990; 23–27.

[2] Kasl, S.V. *Stress and Health. Ann. Rev. Public Health*, 1994; **5**: 3196 341.
[3] Dobson, C.B. *Stress: The Hidden Adversary*. Lancaster, England, MTO Press Ltd. International Medical Publishers, 1982.
[4] Lazarus, R.S. *International Encyclopedia of Social Science*, 1968.
[5] Selvamurthy, K., Sridharan, and Chaudhuri, B.N. *Stress Physiology Proceeding of the National Symposium on Stress Physiology*, New Delhi, September 1986.
[6] Schaierer, A. Use of relaxation sound tracks in the dental Office. *Zahnarztl prax*, 1991; **42**(8): 286–8.
[7] Shakula, A.V. The efficacy of restoring the professional health of flight personnel in a combat unit. *Vrach Delo*, 1994; **7–8**: I49–52.
[8] Gauthier, P.A. and Dallaire, C. Music therapy, *Can. Nurse* 1993; **89**(2): 46–8.
[9] Barthel, J.S., Marshall, J.B., King, P.D. *et al.* Patients and procedure factors associated with a negative patients perception of EGD. *10th world Congresses of Gastroenterology*, 1994, Abst. 2413p.
[10] Mastropaolo, G., Luca, M.G., Galwazzi, F. *et al.* Increased patients anxiety levals before endoscopic procedures. *10th World Congresses of Gastroenterology* 1994. Abst. 2378.
[11] Gordon, M.J., Meyer, G.R. and Meyer, G.W.: Topical Lidocaine in preendoscopic. *Gastroenterology* 1976; **71**: 564–569.
[12] Cattan, E.L., Artnak, E.J., Meyer, G.W. *et al.* Efficacy of atropine as an endoscopic pre-medication *Gastrointest. Endosc.* 1981; **27**: 120.
[13] Gastiglioni, L.J., Allen, Ts. and Patterson, M.: Intravenous diazepam, an improvement in pre-endoscopic medication. *Gastrointest. Endosc.* 1973; **19**: 134–136.
[14] Gordon, M.J., Meyes, G.R., Landsbaum, C.J. *et al.* Mepridine and topical lidocaine in preendoscopic medication. *Gastrointest. Endosc.* 1977; **24**: 14–16.
[15] Kumagai Yoshiya., Makuuchi Hiroyasu. Practical fiberoptic esophagoscopy, IGAKU-SHON, Tokyo–New York, First edition, 1987.
[16] Claudia Wallis. Faith and healing, *Time* 24 June 1996; pp. 39–41.
[17] Rajneesh Bhagwan Shree. *Meditation. The First and Last Freedom*. First Edition. The Publishing House GmbH, Cologne, West Germany.
[18] Chopra Depak, *Music as Medicine, Perfect Health the Complete Mind/Body Guide*, Trans World publishers; 1995; pp. 154–156.
[19] Poldinger, W. and Kocher, R. *Pain in Society and in Medicine*.
[20] Ishii, C., Hagihara, S. and Minamisawa, R. Effects of music on relieving pain associated with a compulsory posture, *Nihon Kango Kagakkaishi*, 1993; **13**(I): 20–7.
[21] Whipple, B. and Glynn, N.J. Quantification of the effects of listening to music as a non invasive method of pain control, *Sch. Inq. Nurs. Pract.* 1992 Spring; **6**(i): 43–58, discussion 59–62.
[22] Brachili, P. Comparative study of the psychophysiologic relaxation effects of an optic–acoustic mind machine with relaxation music. *Z. Exp. Angew. Psychol.* 1993; **40**(2): 179–93.
[23] Fried, R. Integrating music in breathing training and relaxation: I. Background, rationale, and relevant elements. *Biofeedback self regul.* 1990; **15**(2): 161–9.
[24] Mockel, M., Strok, T., Vollwrt, J. *et al.* Stress reduction through listening to music; effects on stress hormones, hemo dynamics and mental state in patients with arterial hypertension and in healthy person. *Dtsch. Med. Wochenscher.* 1995; **120**(21): 745–52.
[25] Mynchenber, T.L. and Dungan, J.M., A relaxation protocol to reduce patient anxiety. *Dimens. Crit.* Care Nurs. 1995; **14**(2): 78–85.
[26] Escher, J., Homann, U. and Anthenien, L. Music during gastroscopy. *Shweiz. Med. wochenschr.* 1993; **123**(26): 1354–8.
[27] Escher, J., Hohmann, U. and Wasem, C. Music therapy and internal medicine. *Schweiz. rundsch. med. prax.* 1993; **82**(36): 957–63.
[28] Borchgrevirk, H.M., Music. brain and medicine, *Tidsskr Nor leegeforen* 1993; **113**(30): 3743–7.
[29] Tang, C.S., Ko Cj, Ng *et al.* Walkman music during epidural anesthesia, *Kao Hsiung I Hseh. KO Hsuesh. Tsa. Chih.* 1993; **9**(8): 468–75.
[30] Rasco, C. Using music therapy as distraction during lumbar punctures (see comments). *J. Pediatr. oncol. Nurs.* 1992; **9**(I): 33–4.
[31] Engelmann, I. Music therapy in phychiatric clinics. Questionnaire on utilization and relization. *Nervenarze* 1995; **66**(3): 217–24.
[32] Bollea, E. and Guarino, A. Group music therapy in the comprehensive management of psychosis. *Minerva Psichiatr.* 1991; **32**(2): 109–17.
[33] Raker, T. Music therapy evaluated by schizophrenic patients. *Psychiatr prax.* 1991; **18**(6): 216–21.
[34] Wilhemson-Lindell, B. Music psychotherapy. *Lakartidningen* 1990; **87**(17): 1471–3.
[35] Baranger, J.P., Charles, L. and Toraine, M. Listen to music therapy. *Soins. Psychiatr.* 1990; **113**: 22–4.
[36] Courtright, P., Johnson, S., Baumgartner, M.A. *et al.* Dinner music does it effect the behavior of psychiatric inpatients? *J. Psychosoc. Nurs; Ment. Health Serv.* 1990; **28**(3): 37–40.
[37] Steinberg, R., Kimming, V. and Raith, L. Music psychopathology IV. The course of musical expression during music therapy with psychiatric in patients. *Psychopathology* 1991; **24**(3): 121–9.
[38] Mita, T., Nakit, Murakami, M. *et al.* An attempt to prevent Psychological distress induced by magnetic resonance imaging; effectiveness of psychological interventions. *Seishin Shinkeigaku Zasshi* 1995; **97**(I): 64–72.
[39] Slifer, K.J., Penn Jones, K. and Cataldo, M.F. Music enhances patient's comfort during MR imaging (Letter) *AJR AM J. Roentgenol.* 1991; **156**(2): 403.
[40] O'Callaghan, C.C. Communicating with brain-imoaired palliative care patients through music therapy. *J. Palliat. Care* 1993; **9**(4): 53–5.
[41] Jochims, S. Coping with illness in the early phase of severe-neurologic diseases. A contribution of music therapy to psychological management in selected neurologic disease pictures. *Psychother. Psychosom. Med. Psychol.* 1990; **40**(3–4): ii5–22.
[42] Oldfild, A. and Adams, M. The effect of music therapy on a group of profoundly mentally handicapped adult. *J. Ment. Defic. Res.* 1990; **34**(Pt2): 107–25.
[43] Sakata, A. Assisting disabled elderly people to live to the best of their abilities. Music therapy for the elderly with dementia at belland general Hospital in Osaka. *Kngo* 1992; **44**: 2037–46.
[44] Standley, J.M. and Hanser, S.B. Music therapy research and application in pediatric oncology treatment. *J. Pediatr. Oncol. Nurs.* 1995; **12**(I): 3–8, Discussion 9–10.

[45] Winter, M.J., Paskin, S. and Baker, T. Music reduces stress and anxiety of patients in the surgical area. *J. Ost. Aesth. Nurs.* 1994; **9**(6): 340–3.

[46] Stevens, K. Patients' Perceptions of music during surgery. *J. ADV. Nurs.* 1990; **15**(9): Io45–51.

[47] Steelman, V.M. Intraoperative music therapy. Effects of anxiety, blood pressure. *AORN J.* 1990; **52**(5): 1026–34.

[48] Hicks, F. The role of music therapy in the care of the newborn. *Nurse Times* 1995; **91**(38): 31–3.

[49] Dardart, E. Touch and music in neonatology or the maintenance of the parent-child bond. *Rev. Infirm.* 1992; **42**(9): 24–8.

[50] Mornhinweg, G.C. and Voigner, R.R. Music for sleep disturbance in the elderly. *J. Holist. Nurs.* 1995; **13**(3): 248–54.

[51] Mandel, S.E. The role of the music therapist on the hospice/palliative care team. *J. Palliat. Care* 1993; **9**(4): 37–9.

[52] Porchet-Munros. Music therapy perspectives in palliative care education. *J. Palliat. Care* 1993; **9**(4): 39–42.

[53] White, J.M. Music therapy: an intervention to reduce anxiety in the myocardial infarction patient. *Clin. Nurse Spec.* 1992; **6**(2): 58–63.

[54] Beck, S.I. The therapeutic Use of music for cancer-related pain. *Oncol. Nurs. Forum* 1991; **18**(8): 1327–37.

[55] Bolwerk, C.A. Effects of relaxing music on state anxiety in myocardial infarction patients. *Crit. Care Q.* 1990; **2**: 63–72.

[56] White, J. and Shaw, C. Music therapy; a means of reducing anxiety in the myocardial patients. *Wis. Med. J.* 1991; **90**(7): 434–7.

[57] Randall, T. Music not only has charms to soothe, but also to aid elderly in coping with various disabilities (News) *JAMA* 1991; **266**(IO): 1323–4, 1329.

[58] Lane, D. Music therapy; a gift beyond measure. *Oncol. Nurs. Forum.* 1992: **I**(4): 178–9.

[59] Beck, S.I. The therapeutic use of music for cancer-related pain. *Oncol. Nurs. Forum.* 1991; **18**(8): 1327–37.

[60] Kerkvliet, G.J. Music therapy may help control cancer pain (NEWS). *J. Natl. Cancer Inst.* 1990; **82**(5): 350–2.

[61] Biley, F. Complimentary therapy: Using music in hospital settings. *Nurs. Stand.* 1992; **6**(35): 37–9.

[62] Hamer, B.A. Music therapy: Harmony for change. *J. Psychosoc. Nurs. Ment. Health Serv.* 1991; **29**(12): 5–7.

[63] Fried, R. Integrating Music in breathing training and relaxation: II. *Application, Biofeed Back Self Regul* 1990; **15**(2): 171–7.

[64] Glyan, N.J. The Music therapy assessment tool in Alzheimer's patients. *J. Gerontol. Nurs.* 1992; **18**(i): 3–9.

[65] O' Sullivan, R.J. A musical road to recovery; Music in intensive care. *Intensive Care Nurs.* 1991; **7**(3): 160–3.

[66] Updika, P. Music therapy results for ICU patients, *Dimens. Crit. Care Nurs.* 1990; **9**(I): 39–45.

[67] Heitz, L., Symreng, T. and Scamman, F.L. Effect of music therapy in the post anesthesia care unit; a nursing intervention.

[68] Mornhinweg, G.C. Effects of music preference and selection in stress reduction. *J. Holist. Nurs.* 1992; **10**(2–101–9).

[69] Frandsen, J.L. Music is a valuable anxiolitic during local and regional anesthesia (Editorial) *Nurse Anesth.* 1990; **1**(4): 181–2.

